# Effects of economic regional differences and family on adolescents’ aggressive behaviors: Perspective of ecosystem integration

**DOI:** 10.1002/brb3.2856

**Published:** 2022-12-27

**Authors:** Xinwei Hong, Shen Liu, Hang Fan, Hongwei Xie, Shengjie Fang, Lin Zhang

**Affiliations:** ^1^ Department and Institute of Psychology Ningbo University Ningbo P. R. China; ^2^ Department of Psychology School of Humanities and Social Sciences, Anhui Agricultural University Hefei P. R. China; ^3^ Department of Psychology Renmin University of China Beijing P. R. China; ^4^ Qibao No. 2 Middle School Shanghai P. R. China

**Keywords:** aggressive behaviors, family socioeconomic status, parenting styles, peer relationship, psychological capital

## Abstract

**Introduction:**

Based on the ecological systems theory and cumulative risk model, the current study aimed to determine the mediating effects of parenting styles, peer relationship, and psychological capital on family socioeconomic status and adolescents’ aggressive behaviors, as well as the moderating effects of economic regional differences.

**Methods:**

In a cross‐sectional design, 1271 Chinese adolescents were recruited to complete the MacArthur Scale, the short‐form Egna Minnen av Barndoms Uppfostran, the Positive PsyCap Questionnaire, the peer support subscale in the Student Personal Perception of Classroom Climate, and the Aggression Questionnaire.

**Results:**

After controlling for gender and age, parenting style, peer relationship, and psychological capital not only mediated, but also constituted multiple chains mediation between family socioeconomic status and aggressive behaviors. Moreover, economic regional differences moderated the multiple chains mediation model between family socioeconomic status and aggressive behaviors.

**Conclusion:**

The accumulation of multiple adverse factors increases the probability of inducing aggressive behaviors, and the development of psychological capital helps reduce the occurrence of aggressive behaviors in adolescents.

## Graphical abstract

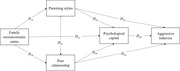

## 1 INTRODUCTION

In 2021, a report released by the World Health Organization indicated that violence was the fourth leading cause of death among adolescents and young people in the world, especially in low‐income and middle‐income countries in the Americas region, with nearly one‐third of male adolescents worldwide dying of violence. Research has confirmed that adolescents in low‐income communities (Hamner et al., 2015) and rural areas (Ma et al., 2020) exhibit more aggressive behaviors, which suggests that a low socioeconomic status (SES) and rural living conditions can create an adverse social environment that leads to aggressive behaviors. However, most existing studies focus on microsystems such as those of parents and peers (Khoury‐Kassabri et al., 2020; Sun & Sun, 2021), and the effect of economic and cultural conditions on the development of adolescents’ social behavior. Few studies have investigated the theoretical integration of the effects of economic and cultural levels, as well as family and peer environment, and its resulting impact on individual development. Therefore, within the framework of the ecological systems theory and cumulative risk model, the current study intends to (1) focus on macrosystem factors (economic regional differences), exosystem factors (family SES), microsystem factors (parenting styles and peer relationship), and individual factors (psychological capital); (2) clarify the functional relationship between various systems and their levels; (3) explore the occurrence mechanism of adolescents’ aggressive behaviors; and (4) determine the conditions necessary for individual differences in aggressive behaviors.

According to the ecological systems theory and cumulative risk model, the development of adolescent behavior is affected by multiple systems, including the social environment, family environment, and peers in which risk factors often co‐occur (D. P. Li et al., 2016). The co‐occurrence of risk factors in multiple systems constitutes a cumulative ecological risk and affects adolescents’ aggressive behaviors (Evans et al., 2013). As the exosystem in the family environment, family SES affects all aspects of the development of children and adolescents (Bradley & Corwyn, 2002) and comprises two aspects: the subjective family SES emphasizes the subjective perception of the individual regarding their social hierarchy (Aydin &Vera, 2020) and the objective family SES indicates family resources received by the individual, for which commonly used measurement indicators include education level, occupation, and family income (Zwar et al., 2020). Studies have shown that disadvantaged family SES can predict adolescents’ aggressive behaviors (B. Chen, Zuo, et al., 2018; Prendergast & MacPhee, 2020). However, the relationship between family SES and adolescent aggressive behaviors requires further exploration.

Parents are the first teachers of their children, and their parenting styles have a significant influence on adolescents’ aggressive behaviors. Arrindell et al. (1999) divided parenting styles into emotional warmth, overprotection, and rejection. Among these parenting styles, emotional warmth can serve as means of positive education to effectively reduce aggression (Masud et al., 2019), whereas negative parenting styles contribute to aggressive behaviors in children and adolescents (Mukhtar & Mshmood, 2018). According to the family stress model, a family's daily material needs will not be met in a low SES household, which will cause the individual to experience psychological stress and hinder the parents’ healthy moods and parenting styles (Gudmunson et al., 2007). Rubin and Kelly (2015) also reported that family SES affects parenting styles. According to the ecological systems theory, the exosystem (family SES) can only act on adolescents through microsystems (parenting styles) (Bronfenbrenner, 1992). Therefore, parenting styles are often used as mediators between family SES and social behavior. For example, Boe et al. (2014) found that parenting styles mediated between family SES and externalizing behavior. Accordingly, parenting styles may play a mediating role between family SES and adolescents’ aggressive behaviors.

The peer group is another important microsystem that significantly impacts adolescents’ psychological development and behavior. Negative peer relationship (e.g., peer victimization) makes adolescents feel hostile and repulsive to their peers, making them more willing to find and establish friendships with deviant peers. Friendships with deviant peers are an important risk factor for aggressive behaviors among adolescents (McQuade & Amherst, 2017). Adolescents with high levels of peer support receive more emotional support, resolve conflicts with others in a reasonable way, and show less aggressive behaviors than those with low levels of peer support (Sanchez‐Moscona & Eiroa‐Orosa, 2021). According to the family stress model, low family SES increases parental stress, which, in turn, triggers negative emotions such as depression and affects children's physical and mental development and environmental adaptability (Conger et al., 2010). Adolescents with low family SES have relatively poor growth environments and may have distortions in personality development. They are often more pessimistic and hostile, lack a sense of control, cannot successfully manage relationships with peers (J. Li et al., 2020), and are prone to aggressive behaviors. Accordingly, peer relationship may mediate the relationship between family SES and adolescents’ aggressive behaviors.

While discussing risk factors for the development of adolescents, we must also pay attention to their ability to actively seek out and cultivate protective factors that can mitigate accumulated risks. Psychological capital is an important individual protective factor for adolescents; it provides the psychological energy and resources to buffer the impact of adverse environments (Wang et al., 2017). Psychological capital can be defined as states or traits, and state‐like psychological capital includes the four elements of optimism, hope, self‐efficacy, and resilience (Luthans et al., 2007). Previous studies have shown that optimism and self‐efficacy are positive human psychological resources that can buffer the adverse effects of life stress and reduce the occurrence of aggressive behaviors (Coneo et al., 2016; Hadley et al., 2015). For example, Valois et al. (2017) found that self‐efficacy can negatively predict aggressive behaviors. In addition, according to the state‐like theory of psychological capital and previous research, psychological capital as a synthesis is not only a stable trait but also a state subject to changes in environment and time (Luthans et al., 2007). A high family SES can provide children and adolescents with abundant material resources and emotional support. When adolescents have sufficient resources, they often have better coping skills, experience a higher level of self‐efficacy, and have an optimistic attitude (Ouyang & Fan, 2018). Accordingly, psychological capital may mediate the relationship between family SES and adolescents’ aggressive behaviors.

According to the ecological system theory, disadvantaged family SES is a remote environmental risk factor that influences individual development (psychological capital, aggressive behaviors) through the microsystem (parenting styles, peer relationship), that is, the microsystem mediates the relationship between the exosystem and individual development. Studies have found that the psychological capital of senior high school students is significantly positively correlated with positive parenting styles (e.g., emotional warmth) and significantly negatively correlated with negative parenting styles (e.g., overprotection and denial) (M. X. Xu, Xu, et al., 2021). Children's psychological capital is improved if parents treat their children warmly and with understanding. Similarly, previous studies have shown that positive peer relationship can directly predict the psychological capital of adolescents and provide them with opportunities to obtain greater psychological capital (Wu et al., 2021). In summary, both families and peers can improve the level of psychological capital of adolescents. In addition, the ecological system theory suggests that the interaction between an individual and the environment influences the development of individual behavior (Bronfenbrenner, 1992). Parents with high SES face less pressure in their lives. They raise their children with positive parenting styles that promote positive emotions and affective experiences for children, which in turn cultivates positive behaviors for children (S. L. Luo et al., 2021). Conversely, parents with low family SES are busy with work and lack supervision of their children and the necessary parent–child communication; consequently, adolescents are easily rejected by their peers, which is not conducive to the development of their psychological capital and leads to various problematic behaviors (X. L. Luo & Li, 2015). Therefore, family SES may influence psychological capital through parenting styles and peer relationship, ultimately affecting adolescents’ aggressive behaviors.

The family and peers, as two important subsystems affecting the development of adolescents, are interconnected and do not develop independently. The exposure of adolescents to the risk factors in one subsystem will increase their exposure to the risk factors in the other subsystem, which will increase the likelihood of their delinquent behaviors (Song et al., 2017). Research has established that parenting styles can effectively predict peer relationship among children and adolescents (Augustine & Stifter, 2015). For example, McDowell and Parke (2009) found that parents who adopted positive parenting styles were able to give children more emotional support, making them more generous and empathetic during peer interactions, which led to peer group acceptance and positive peer relationship. However, children who grew up with negative parenting styles tended to use authoritarian, punitive, and other interpersonal communication strategies and often suffered rejection from their peers, which led to worsening peer relationship that was often characterized by violent behaviors (H. Xu, Zhang, et al., 2008). Simultaneously, based on the previous theories and literature review, it can be inferred that parents with low family SES often adopt negative parenting styles such as rejection and coddling, which makes it difficult for adolescents to identify negative interpersonal communication patterns and form harmonious peer relationship. Therefore, negative parenting styles are not conducive to the cultivation and development of psychological capital, which, in turn, leads to an increase in aggressive behaviors.

Although China's society and economy have developed rapidly, there are still large differences in economy, culture, etc., between regions and urban areas versus rural areas due to geographical location and policy. The imbalance in economic and cultural development across various regions may cause corresponding differences in the development and behaviors of adolescents. A meta‐analysis of violent behavior indicated that the factors influencing youth violent behavior in developing countries are similar to those in developed countries, and include poor parental supervision, parent–child conflict, low family SES, and deviant peer affiliation (De Ribera et al., 2019). However, the overall level and incidence of violence among adolescents in developing countries remain higher than among those in developed countries (Murray et al., 2013). Given the paucity of previous research on the interaction of economic regional differences with family SES, parenting styles, peer relationship, and psychological capital, we conducted an exploratory analysis of the moderating effect of economic regional differences and did not formulate specific hypotheses.

Summing up, family SES is expected to be associated with adolescents’ aggressive behaviors through the mediating role of parenting styles, peer relationship, and psychological capital. Besides, economic regional differences are assumed to moderate the extent to which family SES predicts adolescent aggressive behaviors.

### 1.1 The current study

**FIGURE 1:**
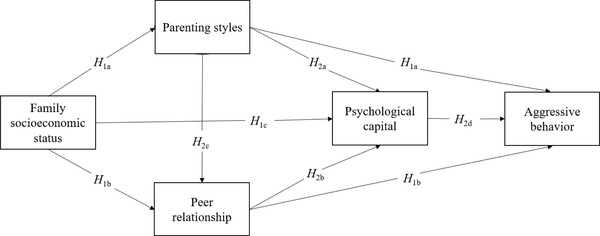
Hypothesized research model (since all path are moderated, the hypothesis *H*_3_ is omitted from the figure)

We propose a moderated multiple chains mediation model (see Figure 1) to explore the roles of parenting styles, peer relationship, psychological capital, and economic regional differences in the relationships between family SES and adolescents’ aggressive behaviors. The hypotheses are as follows:

*H*_1_ *_a_*: Parenting styles play a mediating role between family SES and adolescents’ aggressive behaviors.

*H*_1_ *_b_*: Peer relationship plays a mediating role between family SES and adolescents’ aggressive behaviors.

*H*_1_ *_c_*: Psychological capital plays a mediating role between family SES and adolescents’ aggressive behaviors.

*H*_2_ *_a_*: Parenting styles and psychological capital play a chain‐mediating role between family SES and adolescents’ aggressive behaviors.

*H*_2_ *_b_*: Peer relationship and psychological capital play a chain‐mediating role between family SES and adolescents’ aggressive behaviors.

*H*_2_ *_c_*: Parenting styles and peer relationship play a chain‐mediating role between family SES and adolescents’ aggressive behaviors.

*H*_2_ *_d_*: Parenting styles, peer relationship, and psychological capital act as multiple chains mediating the relationship between family SES and adolescents’ aggressive behaviors.

*H*_3_: Economic regional differences moderate the extent to which family SES predicts adolescent aggressive behaviors.

## 2 METHOD

### 2.1 Participants

Based on the GDP per capita ranking of each province and city in China in 2020, Shanghai ranked second and Anhui Province ranked 13th in the country. Questionnaire surveys were conducted for two middle schools in Anhui Province (representing economically underdeveloped areas) and Shanghai (representing economically developed areas) using the cluster random sampling method. A total of 1300 adolescents participated in the current study. Twenty‐nine cases were removed from the analysis due to their inconsistent responses. The final sample included 1271 participants, 665 boys (52.3%) and 606 girls (47.7%), aged between 10 and 19 years (*M* = 14.64, SD = 1.96). Among the participants, 254 (20%), 218 (17.2%), and 205 (16.9%) adolescents were in junior first, second, and third grades, respectively; 251 (19.7%), 215 (16.9%), and 128 (10.1%) adolescents were in senior first, second, and third grades, respectively. A total of 677 (53.3%) and 594 (46.7%) were from economically developed areas and economically underdeveloped areas, respectively. The participants were informed that their participation in the study was voluntary and that their responses would be treated in confidence. All subjects gave written informed consent in accordance with the Declaration of Helsinki. They were free to withdraw during the research process, and their privacy and personal information were respected and kept confidential.

### 2.2 Measurements

#### 2.2.1 Family SES

The MacArthur Scale was used to measure subjective family SES and included one item. The item contained a ladder with 10 levels that ranged from low to high and represented the social position of families. A higher rating indicated a higher subjective SES (Adler et al., 2000). Participants assessed the level of the ladder according to their situation.

The objective family SES was measured by items assessing family income, education level, and occupation. Referring to the method of B. Chen and Zhao (2017), the current study investigated the education level of the participants’ parents (e.g., “What is your father's/mother's education?”), occupation (e.g., “What is your parent's/mother's occupation?”), and average monthly income (e.g., “what is your father's/mother's average monthly income?”). The measure of objective family SES comprised six items rated using a seven‐point scale, with three items for father and mother. The total score of the six items was calculated as an indicator of objective family SES. Higher scores indicated a higher objective family SES.

#### 2.2.2 Parenting styles

The short‐form Egna Minnen av Barndoms Uppfostran was revised by Jiang et al. (2010) to assess parenting styles. The instrument comprised 42 items, which were separated into father and mother subscales of 21 items each. It included three dimensions: parental refusal (e.g., “My father/mother often criticizes me in front of others for being lazy and useless”), overprotection (e.g., “I feel that my father/mother interferes with me in doing anything”), and emotional warmth (e.g., “Father/Mother praise me”). The items were rated from 1 (never) to 4 (always). The items in the emotional warmth dimension were reverse‐scored, according to the scoring method of Z. F. Peng et al. (2020) and by considering the parenting styles of fathers and mothers. Higher scores indicated more negative parenting styles. In the current study, the internal consistency of the total questionnaire and the three dimensions was 0.91, 0.91, 0.80, and 0.85, respectively.

#### 2.2.3 Psychological capital

The Positive PsyCap Questionnaire (PPQ) was compiled by K. Zhang et al. (2010) to assess psychological capital and contains 26 items. It includes four dimensions: optimism (e.g., “I can always see the good side of things”), hope (e.g., “I am positive, study and work hard to achieve their ideals”), self‐efficacy (e.g., “Many people appreciate my talents”), and resilience (e.g., “I can quickly recover when I encounter a setback”). The items were rated from 1 (very inconsistent) to 7 (very consistent). Higher scores indicated higher levels of PsyCap. In the current study, the internal consistency of the total questionnaire and the four dimensions were 0.91, 0.76, 0.74, 0.77, and 0.81, respectively.

#### 2.2.4 Peer relationship

The peer support subscale in the Student Personal Perception of Classroom Climate (SPPCC) was compiled by Rowe et al. (2010) to measure peer relationship and contains eight items. A representative item was “You received support and care from classmates or friends.” The items were rated from 1 (none) to 4 (completely supported). Higher scores indicated better peer relationship. In the current study, the internal consistency of the subscale was 0.78.

#### 2.2.5 Aggressive behaviors

The Aggression Questionnaire was compiled by Buss and Perry (1992) to assess aggressive behaviors and contains 29 items. It includes four dimensions: physical aggression (e.g., “If anyone hits me, I will fight back”), verbal aggression (e.g., “When others disagree with me, I can't help but argue with them”), anger (e.g., “Sometimes I get angry for no reason”), and hostility (e.g., “Sometimes I think someone is laughing at me behind my back”). The items were rated from 1 (very inconsistent) to 5 (very consistent). Higher scores indicated more aggressive behaviors. In the current study, the internal consistency of the total questionnaire and the four dimensions was 0.85, 0.75, 0.60, 0.67, and 0.79, respectively.

### 2.3 Procedure and data analysis

After obtaining informed consent from the school leaders and the students themselves, the test was administered as a group in a classroom setting by postgraduate psychology students. After the instructions were explained, the participants independently completed all questions in the questionnaire. It took about 20 min to complete the questionnaire, and all questionnaires were distributed and collected in one sitting.

Before conducting the analyses, all variables were transformed into *z*‐scores to allow the computation of standardized coefficients. Correlation and regression analyses were performed using SPSS 22.0. The PROCESS macro (Model 6 and Model 92) version 3.3 (Hayes, 2013) was used to estimate the indirect and moderating paths using bootstrapping in SPSS. Serial mediation analysis permits the estimation of direct and indirect effects of an independent variable (*X*) on a dependent variable (*Y*) while modelling a process in which the independent variable cause in mediator 1 (*M*1), which, in turn, causes in mediator 2 (*M*2) and mediator 3 (*M*3), concluding with the dependent variable as the outcome (Hayes, 2013). Model 6 in PROCESS 3.3 allows the control of the indirect effects of each mediator while controlling for other variables, permitting also independent mediator effects analysis, and providing regression coefficients for the causal steps of the specified indirect effects. In addition, Model 92 in PROCESS 3.3 tested whether the moderating variable acted as a moderator on every path. To explain the moderated effect in more detail, the simple slope test was conducted for the significant interactive effect.

The model was run with 5000 bootstrap samples, and the significance of indirect effects was evaluated by examining the bias‐corrected 95% confidence intervals and the normal theory tests of significance. Preacher and Hayes (2008) recommend bootstrapping because it is the most “powerful and reasonable method of obtaining confidence limits for specific indirect effects under most conditions.”

## 3 RESULTS

### 3.1 Descriptive statistics

The results of the descriptive statistics and correlation analyses are shown in Table 1. Among the demographic variables, gender was negatively correlated with aggressive behaviors (*r* = −0.06, *p* < .05), and age was positively correlated with aggressive behaviors (*r* = 0.09, *p* < .01). Therefore, gender and age were used as control variables in the subsequent analyses. In addition, there were significant correlations between economic regional differences, family SES, parenting styles, peer relationship psychological capital, and aggressive behaviors.

**TABLE 1:** Means, standard deviations, and correlation matrices of study variables (N = 1271)

	*M*	SD	1	2	3	4	5	6	7	8
1. Gender	0.48	0.50	–	–						
2. Age	14.64	1.96	–	–						
3. Economic regional differences ^b^	0.53	0.50	–	–	–					
4. Objective family SES	25.39	7.92	−0.03	−0.67***	0.77***	–				
5. Subjective family SES	5.07	1.74	−0.01	−0.40***	0.48***	0.58***	–			
6. Parenting styles	79.69	16.16	−0.07*	0.04	−0.06*	−0.12***	−0.15***	–		
7. Peer relationship	23.37	4.03	0.02	−0.12***	0.14***	0.20***	0.23***	−0.37***	–	
8. Psychological capital	124.52	23.33	−0.08**	−0.13***	0.14***	0.21***	0.24***	−0.43***	0.52***	–
9. Aggressive behavior	73.72	15.60	−0.06*	0.09**	−0.11***	−0.11***	−0.11***	0.33***	−0.31***	−0.43***

*Note*. Gender is a binary variable; 0 = boys, 1 = girls; economic regional differences are a binary variable: 0 = economically underdeveloped areas, 1 = economically developed areas.

Abbreviation: SES, socioeconomic status.

* *p* < .05

** *p* < .01

*** *p* < .001, the same as below.

### 3.2 Test of the multiple chains mediation model

The results of the correlation analysis for parenting styles, peer relationship, and psychological capital met the statistical requirements for a mediation analysis (Wen & Ye, 2014). The results of the regression analysis showed that the objective and subjective family SES significantly predicted parenting styles (*p*s < .001), peer relationship (*p*s < .001), and psychological capital (*p*s < .05). Second, parenting styles significantly predicted peer relationship (*p*s < .001), psychological capital (*p*s < .001), and aggressive behaviors (*p*s < .001). Third, peer relationship significantly predicted psychological capital (*p* < .001) and aggressive behaviors (*p*s < .01). Finally, psychological capital significantly predicted aggressive behaviors (*p* < .001).

**FIGURE 2:**
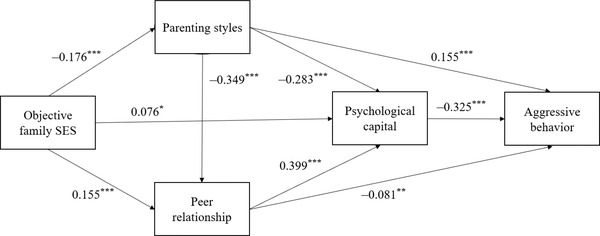
The mediating effect of objective family socioeconomic status (SES)

**FIGURE 3:**
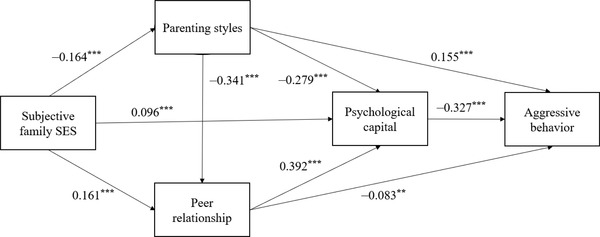
The mediating effect of subjective family socioeconomic status (SES)

The results of the mediating effect analysis showed that parenting styles, peer relationship, and psychological capital significantly mediated the relationship between family SES and aggressive behaviors (see Figures 2 and 3). The mediating effect consisted of seven paths of indirect effects, all of which were significant (as shown in Tables 2 and 3). Thus, parenting styles, peer relationship, and psychological capital partially mediated the relationship between objective and subjective family SES and aggressive behaviors, and constituted a multiple chain mediation model.

**TABLE 2:** Decomposition table of mediating effect (objective family socioeconomic status [SES])

Mediating path	Mediating effect	Standard error	95% Confidence intervals	Effect size (%)
*X*_1_→*M*_1_→*Y*	−0.027	0.008	[−0.044, −0.013]	31.765
*X*_1_→*M*_2_→*Y*	−0.013	0.006	[−0.026, −0.002]	15.294
*X*_1_→*M*_3_→*Y*	−0.025	0.010	[−0.045, −0.006]	29.412
*X*_1_→*M*_1_→*M*_2_→*Y*	−0.005	0.003	[−0.010, −0.001]	6.098
*X*_1_→*M*_1_→*M*_3_→*Y*	−0.016	0.004	[−0.025, −0.009]	18.824
*X*_1_→*M*_2_→*M*_3_→*Y*	−0.020	0.006	[−0.033, −0.010]	23.529
*X*_1_→*M*_1_→*M*_2_→*M*_3_→*Y*	−0.008	0.002	[−0.012, −0.004]	9.412
Total indirect effect	−0.113	0.020	[−0.154, −0.075]	
Direct effect	0.027	0.034	[−0.039, 0.096]	
Total effect	−0.085	0.039	[−0.159, −0.011]	

*Note*. *X*_1_: objective family SES; *M*_1_: parenting style; *M*_2_: peer relationship; *M*_3_: psychological capital; *Y*: aggressive behavior.

**TABLE 3:** Decomposition table of mediating effect (subjective family socioeconomic status [SES])

Mediating path	Mediating effect	Standard error	95% Confidence intervals	Effect size (%)
*X*_2_→*M*_1_→*Y*	−0.025	0.007	[−0.040, −0.013]	29.762
*X*_2_→*M*_2_→*Y*	−0.013	0.006	[−0.026, −0.002]	15.476
*X*_2_→*M*_3_→*Y*	−0.031	0.009	[−0.050, −0.014]	36.905
*X*_2_→*M*_1_→*M*_2_→*Y*	−0.005	0.002	[−0.009, −0.001]	5.952
*X*_2_→*M*_1_→*M*_3_→*Y*	−0.015	0.004	[−0.023, −0.009]	17.857
*X*_2_→*M*_2_→*M*_3_→*Y*	−0.021	0.005	[−0.031, −0.012]	24.000
*X*_2_→*M*_1_→*M*_2_→*M*_3_→*Y*	−0.007	0.002	[−0.011, −0.004]	8.333
Total indirect effect	−0.112	0.017	[−0.150, −0.086]	
Direct effect	0.033	0.028	[−0.022, 0.088]	
Total effect	−0.084	0.030	[−0.144, −0.025]	

*Note*. *X*_2_: subjective family SES; *M*_1_: parenting style; *M*_2_: peer relationship; *M*_3_: psychological capital; *Y*: aggressive behavior.

### 3.3 Test of the moderating effect

Model 92 was used to test the moderating effect of regional economic differences on the multiple chain mediation model. The results showed that the interaction between subjective family SES and economic regional differences significantly predicted parenting styles (*β* = −.24, *p* < .001) and peer relationship (*β* = .15, *p* < .05), while the interaction between parenting styles and economic regional differences significantly predicted aggressive behaviors (*β* = −.20, *p* < .001), and the interaction between psychological capital and economic regional differences significantly predicted aggressive behaviors (*β* = −0.24, *p* < .001). That is, the moderating effect of economic regional differences occurred in four paths: “subjective family SES → parenting styles,” “subjective family SES → peer relationship,” “parenting styles → aggressive behaviors,” and “psychological capital → aggressive behaviors.”

**FIGURE 4:**
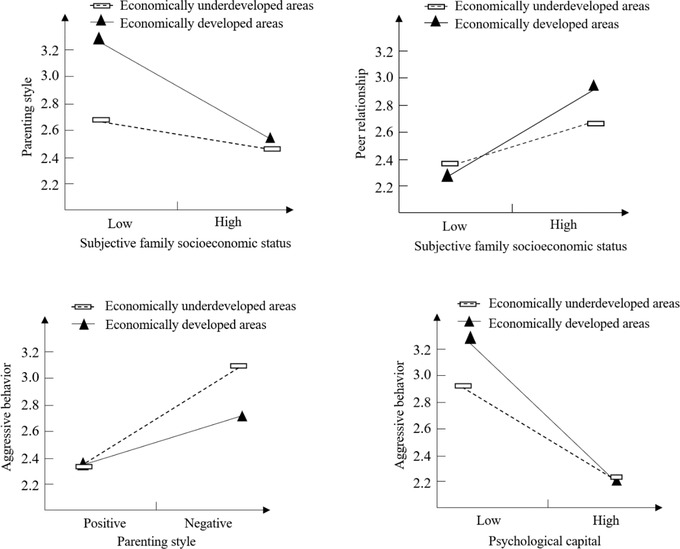
The moderating effect of economic regional differences

To more clearly explain the moderating effect of economic regional differences, the level of regional economic development was divided into economically developed areas and economically underdeveloped areas, and the simple slope was calculated and simple effect analysis plots were created. The results showed that, relative to economically underdeveloped areas, the effect of subjective family SES on parenting styles increased in economically developed areas (from *B*_simple_ = −0.04, *t* = −1.00, *p* = .32 to *B*_simple_ = −0.29, *t* = −6.41, *p* < .001), the effect of subjective family SES on peer relationship increased in economically developed areas (from *B*_simple_ = 0.09, *t* = 2.10, *p* < .05 to *B*_simple_ = 0.24, *t* = 5.54, *p* < .001), while that of parenting styles on aggressive behaviors decreased in economically developed areas (*B*_simple_ = 0.28, *t* = 5.78, *p* < .001 to *B*_simple_ = 0.08, *t* = −2.37, *p* < .05); and the effect of psychological capital on aggressive behaviors increased in economically developed areas (from *B*_simple_ = −0.19, *t* = −4.08, *p* < .001 to *B*_simple_ = −0.43, *t* = −10.62, *p* < .001) (see Figure 4).

## 4 DISCUSSION

The current study found that family SES has a positive effect on adolescents’ aggressive behaviors, confirming the ecological systems theory that the exosystem plays an important role in development. This finding is also consistent with the predictive effect of family SES on aggressive behaviors found in previous studies; the lower the family SES, the higher the aggressive behaviors (Chen et al., 2018; Greitemeyer & Sagioglou, 2016). In contrast with other existing studies (B. Chen & Zhao, 2017), objective family SES had the same predictive validity as subjective family SES on aggressive behaviors in the current study. We speculate that this may be due to the high correlation between subjective and objective family SES, and the relatively small correlation coefficients with parenting styles, peer relationship, psychological capital, and aggressive behaviors, which, in turn, had the same predictive validity for aggressive behaviors.

### 4.1 Independent mediating roles of parenting styles, peer relationships, and psychological capital

First, family SES can predict adolescents’ aggressive behaviors through the independent mediating effects of parenting styles, supporting hypothesis *H*_1a_. There is strong correlation between family SES and parenting styles. Factors such as the parent's occupation and education level not only affect their abilities but also influence their parenting styles and behaviors. According to the family stress model, parents with low family SES have greater economic pressure and social pressure, which in turn can cause emotional and behavioral problems. Parents usually also transfer pressure to the parenting styles and behavior on their children (Conger et al., 2010). They are more likely to adopt strict parenting styles such as punishment and intimidation, which lead to poor social adjustment of adolescents, and consequently a series of problematic behaviors such as aggressive behaviors (X. Y. Zhang et al., 2017).

Second, family SES can predict adolescents’ aggressive behaviors through the independent mediating effects of peer relationships, supporting hypothesis *H*_1b_. That may be due to the fact that different family SES provides a different environment for interpersonal communication and learning among adolescents. Adolescents with higher family SES have more opportunities to follow their parents to participate in activities, and social interactions and interpersonal interactions are relatively frequent (Q. Q. Liu, Zhou, et al., 2020). They can acquire some knowledge about interpersonal communication in their lives, subtly know how to get along with their peers, and integrate into the peer group more easily (Hosokawa & Katsura, 2017). In contrast, adolescents with low family SES are often unable to participate in group activities and may be isolated in school. Moreover, they have lower level of self‐esteem, are more sensitive to peer rejection, and tend to withdraw and reduce interpersonal intimacy after interpersonal conflict and show more behaviors that disrupt relationships (Bai et al., 2021).

Finally, the findings of the study also confirmed that external environmental variables (family socioeconomic status) work through individual variables (psychological capital), and that the impact on adolescents’ behavior changes with the development of individual characteristics (S. L. Luo et al., 2021), supporting hypothesis *H*_1c_. Parents with lower family SES focus their energy on meeting the basic needs of the family and fail to provide more material and emotional support for adolescents. Adolescents experience the injustice brought about by material deprivation, produce passive cognitive styles and coping styles on actual events, and lack positive evaluations about the future, all of which are not conducive to the development of adolescents’ psychological capital (Jia et al., 2021). At the same time, adolescents with low psychological capital are more inclined to adopt a negative attitude and ways to deal with problems (Jia et al., 2021). They lack self‐confidence and self‐efficacy, and are easy to give up when encountering difficulties or setbacks, thus generating or showing more aggressive behaviors (L. Y. Zhang, 2022).

### 4.2 The Sequential mediating role of parenting styles, peer relationship and psychological capital

In the current study, family SES predicted adolescents’ aggressive behaviors through multiple chains, thereby mediating the effects of parenting styles, peer relationship, and psychological capital. This finding suggests that certain sequential characteristics of environmental and individual factors influence adolescents’ aggressive behaviors. Likewise, the finding supported the view of the ecological systems theory and cumulative risk model, which state that individual development is not determined by a single risk factor but is the result of the interaction between various environmental factors, interpersonal relationships, and individual factors, with different risk factors acting in concert with each other.

Parenting styles and psychological capital mediated the relationship between family SES and aggressive behaviors, supporting hypothesis *H*_2a_. Parents with lower family SES do not have sufficient time and energy to nurture their children due to economic pressure, leaving adolescents feel rejection, indifference, and authoritative pressure in the family environment, which directly leads to a poor parent–child relationship (Q. S. Chen, Kong, et al., 2018). Without the deserved care and love from their parents, adolescents lack positive beliefs about the present and future (Roche et al., 2014). In the face of difficulties and challenges, they appear to lack confidence, pessimism, etc., are unwilling to put in persistence and effort, and prone to show high levels of anger and aggressive behaviors (L. Y. Zhang, 2022).

Peer relationships and psychological capital mediated the relationship between family SES and aggressive behaviors, supporting hypothesis *H*_2b_. Adolescents with high family SES have more opportunities to participate in social activities, learn reasonable communication methods and skills, and actively integrate into peer groups. With improved peer relationship, adolescents receive greater psychological and emotional support from their peers, which promotes their development of psychological capital (Wu et al., 2021). Simultaneously, positive peer relationship increases adolescents’ sense of belonging and identification with the group, class, and school; consequently, they tend to conform to school and class norms and adapt to the school environment (Furrer & Skinner, 2003).

Parenting styles and peer relationships mediated the relationship between family SES and aggressive behaviors, supporting hypothesis *H*_2c_. Middle‐class or higher family SES are more likely to have more verbal communication, more emotionally responsive, more authoritative parenting styles, etc., and are better equipped financially to provide a loving and warm family environment for adolescents (Bradley et al., 2001; X. Y. Zhang et al., 2017). They can observe negative emotions arising in their children and provide comfort and advice to help the children alleviate negative emotions so that they do not turn into problematic behaviors (Yamamoto et al., 2016). Through positive interactions with their parents, adolescents learn social rules and behaviors and develop good habits of respecting and trusting others, which helps them communicate with peers and have good peer relationship in school (L. S. Liu, He, et al., 2020). A good peer climate will reduce the risk of nurturing a bad environment and help avoid aggressive behaviors (Sanchez‐Moscona & Eiroa‐Orosa, 2021).

Parenting styles, peer relationships, and psychological capital mediated the relationship between family SES and aggressive behaviors, supporting hypothesis *H*_2d_. According to the cumulative risk model (Evans et al., 2013), the accumulation of multiple risk factors, such as low family SES, negative parenting styles, and poor peer relationship, creates an environment in which adolescents are exposed to many adverse factors that negatively impact individuals. The accumulation of risk factors limits the development of adolescents’ psychological capital, thus forming a “gradient effect” (D. P. Li et al., 2016). Moreover, insufficient psychological capital leads to a decrease in its protective effect, which makes adolescents prone to negative emotions and increases the probability of aggressive behaviors.

### 4.3 The moderating role of economic regional differences

Economic regional differences moderated the multiple chains mediating the relationship between family SES and aggressive behaviors, supporting hypothesis *H*_3_. Probably the reason is that the economically developed areas, represented by Shanghai, where the government and society focus on education, have more economic and material conditions to invest in family and school education. Parents in economically developed areas are influenced by the entire social environment, even if their family SES is not high; they can receive guidance from the community or professional institutions and have more opportunities to learn and be exposed to advanced educational concepts. In contrast, the relevant family education services in economically underdeveloped areas are often insufficient in providing educational guidance for parents.

In contrast, adolescents in economically developed areas have more opportunities to learn legal knowledge and form legal thinking. Simultaneously, the level of economic development is the basis for school educational resources and mental health education. Economically developed areas can provide more mental health teachers and counseling rooms for mental health education in schools, which, in turn, can provide ways for adolescents with low levels of psychological capital to solve physical and psychological problems such as academic pressure and negative emotions (W. J. Peng et al., 2021).

### 4.4 Implications

In terms of theoretical significance, previous studies on aggression have mainly been based on microsystems, lacking a comprehensive consideration of the exosystem and macrosystem in which adolescents are located. The current study explores the relationship between each hierarchical system and adolescent aggression and its underlying mechanisms, and confirms that there are complex interactions among the macrosystem, the exosystem, and the individual, which to a certain extent remedies the shortcomings of previous studies. This study also further supports and enriches ecological systems theory and the cumulative risk model. Human development is affected by multiple subsystems such as society, family, and peers. Adolescents exposed to multiple environmental risk factors are more likely to have psychological crises, and this will have serious impacts on their behavioral development.

In terms of practical significance, previous studies have mainly made recommendations on how to reduce and decrease adolescents’ aggressive behaviors at the microsystem, such as family, school, and peers. However, the current study suggests that we should combine the macrosystem with microsystem systematically prevent and intervene in adolescents’ aggressive behaviors, and that various actors such as the government, society, and family should cooperate. For economically underdeveloped areas, the government and society should adopt supportive policies or resource‐oriented measures to promote education equity and provide relevant education services for parents with low family SES so that parents learn how to develop positive parenting styles. Although the family SES is relatively difficult to change, parents should strive to establish a positive outlook and attitude toward life, improve the level of communication and assistance between parents and children, and increase effective parent–child interaction and company time, which can not only create a positive and warm growth environment for adolescent, but also promote the development of psychological capital. Schools can provide adolescents with interpersonal skill training so that adolescents with low family SES also have good communication skills, improve peer relationships, and thus affect their psychological capital and aggressive behaviors.

In terms of research methodology, the current study is the first attempt to use a moderated multiple chains mediation model. Compared to previous mediating or moderating model, the moderated multiple chains mediation model is helpful to answer the question of how family SES affects aggressive behaviors and under what conditions it affects aggressive behaviors. Moreover, the model is able to incorporate more variables, reveal the between multiple variables more systematically, has better ecological validity, and reveal the occurrence mechanism of adolescents’ aggressive behaviors.

### 4.5 Limitations and future suggestions

The current study has several limitations that provide opportunities for future research. First, a cross‐sectional study was used in the current study that only collected data from adolescents. With a longitudinal investigation, it is possible to explain the causal relationships between variables. Future research can longitudinally track participants with rigorous experimental designs to observe the changes among adolescents in the entire ecological growth process. Second, the study only examined the influence of combined parenting styles on adolescents’ aggressive behaviors. Future research could examine fathers’ and mothers’ styles separately to further explore the effect of parenting styles on adolescent development. Finally, the study selected one province and one city in economically developed and economically underdeveloped areas. Although the interference of regional differences and other factors on the research results can be controlled to some extent, it is not conducive to the generalizability of the study results, especially in China, which has a vast territory and diverse culture. Future research can expand the regional scope of the sample to validate the research conclusions.

## 5 CONCLUSION

In summary, the current study showed that low family SES is associated with increased adolescents’ aggressive behaviors, and also confirmed the mediating role of parenting styles, peer relationships, and psychological capital between family SES and adolescents’ aggressive behaviors, as well as the moderating role of economic regional differences in the mediation model. On the other hand, government, society, and family need to work together to optimize the environment in which adolescents grow up in order to reduce possible aggressive behaviors. It is recommended to conduct interventional studies to investigate the role of these variables on adolescents’ aggressive behaviors in longitudinal studies.

## CONFLICT OF INTEREST

The authors declare no conflict of interest.

### PEER REVIEW

The peer review history for this article is available at https://publons.com/publon/10.1002/brb3.2856

## Data Availability

Data will be made available upon reasonable request.
